# 1.5-T MR relaxometry in quantifying splenic and pancreatic iron: retrospective comparison of a commercial 3D-Dixon sequence and an established 2D multi-gradient echo sequence

**DOI:** 10.1007/s00330-023-09451-0

**Published:** 2023-02-17

**Authors:** Michaela Plaikner, Lukas Lanser, Christian Kremser, Günter Weiss, Benjamin Henninger

**Affiliations:** 1 Department of Radiology, Medical University of Innsbruck, Anichstraße 35, 6020 Innsbruck, Austria; 2 Department of Internal Medicine, Medical University of Innsbruck, Anichstraße 35, 6020 Innsbruck, Austria

**Keywords:** Spleen, Pancreas, Liver, Iron overload

## Abstract

**Objectives:**

To compare the quantitative measurement of splenic and pancreatic iron content using a commercial 3D-Dixon sequence (qDixon) versus an established fat-saturated R2* relaxometry method (ME-GRE).

**Methods:**

We analyzed splenic and pancreatic iron levels in 143 MR examinations (1.5 T) using the qDixon and a ME-GRE sequence (108 patients: 65 males, 43 females, mean age 61.31 years). Splenic and pancreatic R2* values were compared between both methods using Bland–Altman plots, concordance correlation coefficients (CCC), and linear regression analyses. Iron overload (R2* > 50 1/s) was defined for both organs and compared using contingency tables, overall agreement, and Gwet’s AC1 coefficient.

**Results:**

Of all analyzable examinations, the median splenic R2* using the qDixon sequence was 25.75 1/s (range: 5.6–433) and for the ME-GRE sequence 35.35 1/s (range: 10.9–400.8) respectively. Concerning the pancreas, a median R2* of 29.93 1/s (range: 14–111.45) for the qDixon and 31.25 1/s (range: 14–97) for the ME-GRE sequence was found. Bland–Altman analysis showed a mean R2* difference of 2.12 1/s with a CCC of 0.934 for the spleen and of 0.29 1/s with a CCC of 0.714 for the pancreas. Linear regression for the spleen/pancreas resulted in a correlation coefficient of 0.94 (*p* < 0.001)/0.725 (*p* < 0.001). Concerning iron overload, the proportion of overall agreement between the two methods was 91.43% for the spleen and 93.18% for the pancreas.

**Conclusions:**

Our data show good concordance between R2* values obtained with a commercial qDixon sequence and a validated ME-GRE relaxometry method. The 3D-qDixon sequence, originally intended for liver assessment, seems to be a reliable tool for non-invasive evaluation of iron content also in the spleen and the pancreas.

**Key Points:**

• A 3D chemical shift imaging sequence and 2D multi-gradient echo sequence show good conformity quantifying splenic and pancreatic R2* values.

• *The 3D chemical shift imaging sequence allows a reliable analysis also of splenic and pancreatic iron status*.

• *In addition to the liver, the analysis of the spleen and pancreas is often helpful for further differential diagnostic clarification and patient guidance regarding the iron status*.

## Introduction

Iron disorders show differences in degree or distribution of iron overload between organs, underlying etiologies, and treatment [1]. In the primary form of hereditary hemochromatosis, iron overload mainly affects the liver and, in later stages, the pancreas and the heart [2]. In subjects with primary or secondary iron loading, pancreas iron accumulation was defined as a predictor of cardiac iron overload while an iron-free pancreas virtually precludes increased cardiac iron [3, 4]. While in hereditary hemochromatosis the reticuloendothelial system is iron-deficient [5, 6], in secondary iron hemochromatosis, iron accumulates also in macrophages in the liver, spleen, and bone marrow. Accordingly, genetic hemochromatosis is characterized by low iron content in the spleen, with the exemption of ferroportin loss of function mutations where iron is retained in macrophages and thus in the spleen [7]. In hematologic disorders and dysmetabolic syndromes, splenic iron has been reported to be neither decreased nor increased [8]. Therefore, in the diagnostic workup of patients with iron overload, in addition to quantification of liver iron, the evaluation of other abdominal organs such as the spleen and the pancreas has been recommended as important non-invasive diagnostic workups [9, 10].

Magnetic resonance (MR) R2* relaxometry was established in clinical routine as a reliable method to assess liver iron concentration (LIC). Originally, a variety of different MR sequences and post-processing methods were used, which were frequently individually developed and calibrated by the performing center [4]. Therefore, availability of MR relaxometry for LIC quantification was limited to specialized centers with appropriate expertise, and switching between different methods was found to be not advisable [9]. Meanwhile, different vendors have introduced commercial 3D chemical shift imaging sequences which simultaneously allow the quantification of liver R2* values for LIC quantification together with the quantification of hepatic fat fraction. Usually, these sequences do not necessitate separate off-line post-processing, but automatically provide R2* maps to determine hepatic iron content and proton density fat fraction (PDFF) maps to quantify liver fat content. Thus, these parameters have become easily available for a wide range of users and earlier studies have shown that these 3D chemical shift imaging sequences are a reliable tool for MR hepatic iron assessment and their performance has been proven to be comparable with established relaxometry methods [11]. It is important to note that because the underlying post-processing algorithms mostly rely on a multi-peak fat model of the liver [12–14], these sequences are only meant to be used in the liver. But, as mentioned earlier, apart from the liver, the determination of iron loading in the pancreas or the spleen is also of diagnostic importance. These organs are typically within the 3D acquisition volume used for the liver, so that it would be tempting to obtain also R2* values for the pancreas and the spleen from the same 3D chemical shift imaging data set, which actually has already been proposed in the literature [4, 15]. Unfortunately, it has not yet been validated whether R2* values obtained with the commercial 3D chemical shift imaging sequences for the pancreas or the spleen are reliable.

It was therefore the purpose of our study to compare R2* values obtained for the pancreas and the spleen from a commercial 3D chemical shift imaging sequence with values of an established R2* relaxometry method that does not rely on a liver-specific post-processing model [16].

## Materials and methods

This study was approved by the local institutional review board (Medical University of Innsbruck). We retrospectively evaluated 143 MR examinations in 108 patients with respect to not only hepatic but also splenic and pancreatic iron overload. Patients were referred to our department between 05/2020 and 01/2022 for MR imaging of the upper abdomen including quantification of liver iron. All examinations were carried out on a 1.5-T whole body MR system (MAGNETOM Avanto^Fit^, Siemens Healthcare) using an 18-element body matrix coil and 12–16 elements of the integrated 32-channel spine matrix coil. Scans were performed in supine position and images were acquired in transversal orientation during breath-holds at the end of expiration. For iron quantification, we used two different sequences: a commercial 3D chemical shift imaging sequence (qDixon) and a biopsy-calibrated, fat-saturated 2D multi-gradient echo sequence (ME-GRE) [16]. Parameters for both sequences are given in Table 1.

**Table 1:** MR parameters for both sequences routinely used for liver iron quantification

	ME**-**GRE	qDixon
*Initial TE (ms)*	0.99	2.38
*Number of echoes (n)*	12	6
*Delta TE (ms)*	1.41	2.38
*Max. TE (ms)*	16.5	14.28
*TR (ms)*	200	15.6
*Flip angle (°)*	20	4
*Receive bandwidth (Hz/Px)*	1955	1080
*Total acceleration factor*	-	4
*Matrix (mm)*	128 × 128	160 × 120
*Field of view*	360–380	360–380
*Slice thickness (mm)*	10	3.5
*Number of slices*	2^1^ (two breath-holds)	64 (one breath-hold)
*Fat saturation*	CHESS^2^	Dixon
*Data acquisition/type of sequence (2D/3D)*	2D	3D
*Acquisition time (s)*	16.8	18.51

*TE* echo time, *TR* repetition time

^1^Two different positioned single slices through the upper abdomen

^2^Chemical shift selective fat saturation (as defined by the manufacturer)

The qDixon sequence is based on a 3D multi-gradient-echo acquisition with 6 echoes and uses controlled aliasing undersampling (CAIPIRINHA) [17], which allows acquisition in a single breath-hold. During image calculation, the sequence utilizes advanced inline processing using a multi-peak fat model and a multistep adaptive fitting approach to automatically calculate R2* and PDFF maps without need for further post-processing. Any image-viewing software that allows region of interest (ROI)–based signal intensity measurements can be used for measuring R2* and PDFF values. For the qDixon sequence, default parameters suggested by the vendor were used. Image analysis of the obtained images was performed using our clinical standard picture archiving and communication system (IMPAX; Agfa-Gevaert). One MR experienced radiologist (MP) carefully placed three ROI with a mean area of 50 mm^2^ (diameter approx. 8 mm) within the liver parenchyma, two in the pancreas (body and tail) and one with 140 mm^2^ (diameter approx. 13 mm) in the central spleen always avoiding artifacts, big vessels, and focal lesions.

For the ME-GRE sequence, R2* maps were calculated using a custom-written plugin for ImageJ (Wayne Rasband, National Institutes of Health) by fitting on a pixel-wise basis with a single exponential truncation model [16]. ROI placement on R2* maps was then independently performed on co-registered areas by a physicist (CK) with longstanding experience in MRI post-processing.

### Statistical analysis

For further analysis, R2* values for the liver, pancreas, and spleen obtained with both methods were stored as an Excel worksheet. Statistical calculations were then performed using the R Project for Statistical Computing [18]. Splenic and pancreatic R2* values (1/s) were compared between qDixon and ME-GRE using Bland–Altman plots and concordance correlation coefficients (CCC) were calculated. To test the hypothesis that the obtained bias was equal to zero, a one-sample *t*-test was performed. *p*-values < 0.05 indicated a significant difference form zero. In addition, linear regression analysis was performed by fitting a linear model to the data. Finally, iron overload for the pancreas and the spleen was defined as R2* > 50 1/s. Using contingency tables, the agreement of both methods regarding iron overload classification was determined. Agreement coefficients were given by calculating the percent agreement and to avoid paradoxical kappa values, Gwet’s AC1 coefficient was used with the rel-package for R [19–21].

## Results

Altogether, 143 examinations in 108 patients (65 male, 43 female) with a mean (median; range) age of 61.3 (64.0, 19–88) years were included in the initial analysis.

Due to wrong slice positioning (e.g., the pancreas not included in ME-GRE sequence), poor delimitation of the organ, or intense (motion) artifacts, only 140 examinations with an analyzable spleen and 132 with an evaluable pancreas remained.

### Common data

Table 2 represents the resulting pancreatic and splenic values of all patients using the qDixon sequence and the ME-GRE sequence.

**Table 2:** Pancreatic and splenic values of all patients using both sequences

Organ	Liver		Pancreas		Spleen	
Sequence	qDixon	ME-GRE	qDixon	ME-GRE	qDixon	ME-GRE
R2* (1/s) *mean*	56.49	59.54	33.26	33.55	57.16	59.28
R2* (1/s) *median*	35.44	38.44	29.93	31.25	25.75	35.35
R2* (1/s) *first quartile*	28.88	32.21	23.57	25.88	15.97	19.57
R2* (1/s)* third quartile*	51.94	54.09	38.99	38.3	74.6	69.78
R2* (1/s) *minimum*	20.67	21.57	14	14	5.6	10.9
R2* (1/s) *maximum*	476.67	611.7	111.45	97	433	400.8

### Agreement spleen

**Fig. 1:**
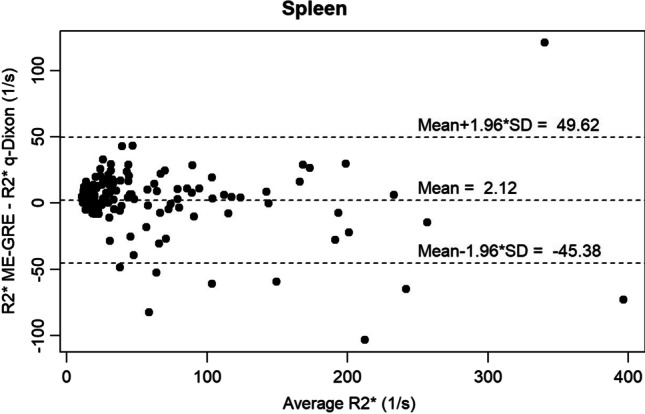
Bland–Altman plot representing the absolute difference of splenic R2* values between qDixon and ME-GRE sequences

Bland–Altman analysis of splenic R2* values between qDixon and ME-GRE resulted in a bias (absolute mean difference) of 2.12 1/s (LoA of 49.62 and − 45.38) with a CCC of 0.934 (0.909–0.952) (Fig. 1). The bias was not significantly different from zero (*p* = 0.302).

**Fig. 2:**
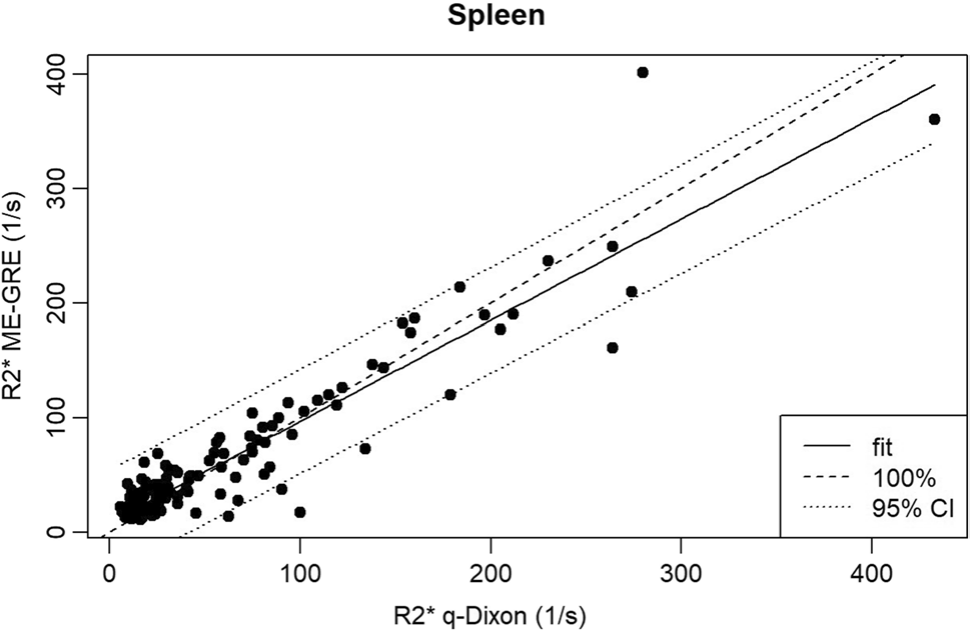
Scatterplot of splenic R2* values of ME-GRE and qDixon

Linear regression analysis correlating splenic R2* values of qDixon and ME-GRE resulted in a correlation coefficient of 0.94 (*p* < 2.2e − 16). The respective scatterplot is shown in Fig. 2.

### Agreement pancreas

**Fig. 3:**
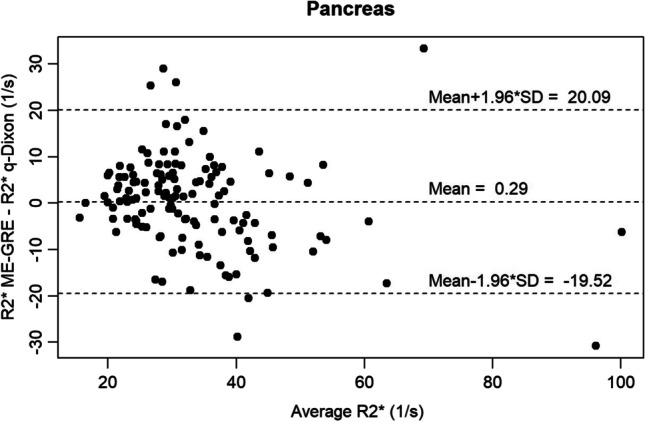
Bland–Altman plot representing the absolute difference of pancreatic R2* values comparing qDixon and ME-GRE sequences

Evaluating the agreement of pancreatic R2* values between qDixon and ME-GRE, the absolute Bland–Altman plot (Fig. 3) showed a mean bias of 0.29 (LoA of 20.09 and − 19.52) with a CCC of 0.714 (0.623–0.786). Again, the bias was not significantly different form zero (*p* = 0.743).

**Fig. 4:**
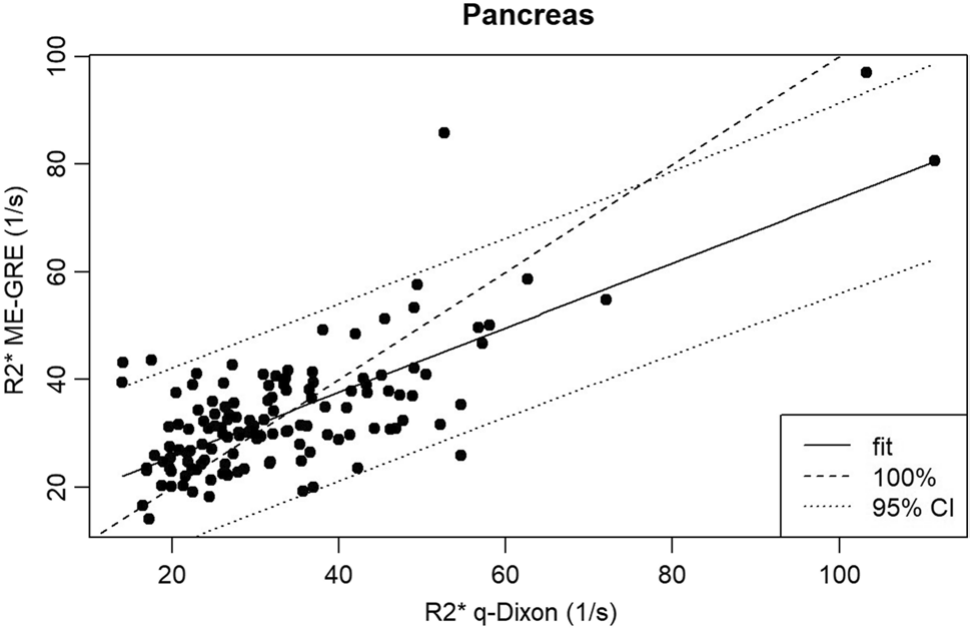
Scatterplot of pancreatic R2* values of ME-GRE and qDixon

Linear regression analysis (Fig. 4) for the pancreas resulted in a correlation coefficient of 0.725 (*p* < 2.2e − 16).

### Analysis regarding iron overload detection

When using a threshold of R2* > 50 1/s for the presence of iron overload in the pancreas or the spleen, the iron assessment in the pancreas agreed in 123 patients between qDixon and ME-GRE and resulted in different ratings (iron overload versus no iron overload) only in 9 patients leading to an overall agreement of 93.18% and a Gwet AC1 of 0.92, indicating strong agreement. For the spleen, iron assessment agreed in 128 and differed in 12 patients, leading to an overall agreement of 91.43% and a Gwet AC1 of 0.844, also indicating strong agreement. The corresponding contingency tables are shown in Table 3.

**Table 3:** Contingency tables of qDixon and ME-GRE examinations regarding splenic (top) and pancreatic (bottom) iron overload

*Spleen*	*qDixon*	
*ME-GRE*	*False*	*True*
*False*	86	6
*True*	6	42
*Pancreas*	qDixon	
*ME-GRE*	*False*	*True*
*False*	117	6
*True*	3	6

**Fig. 5:**
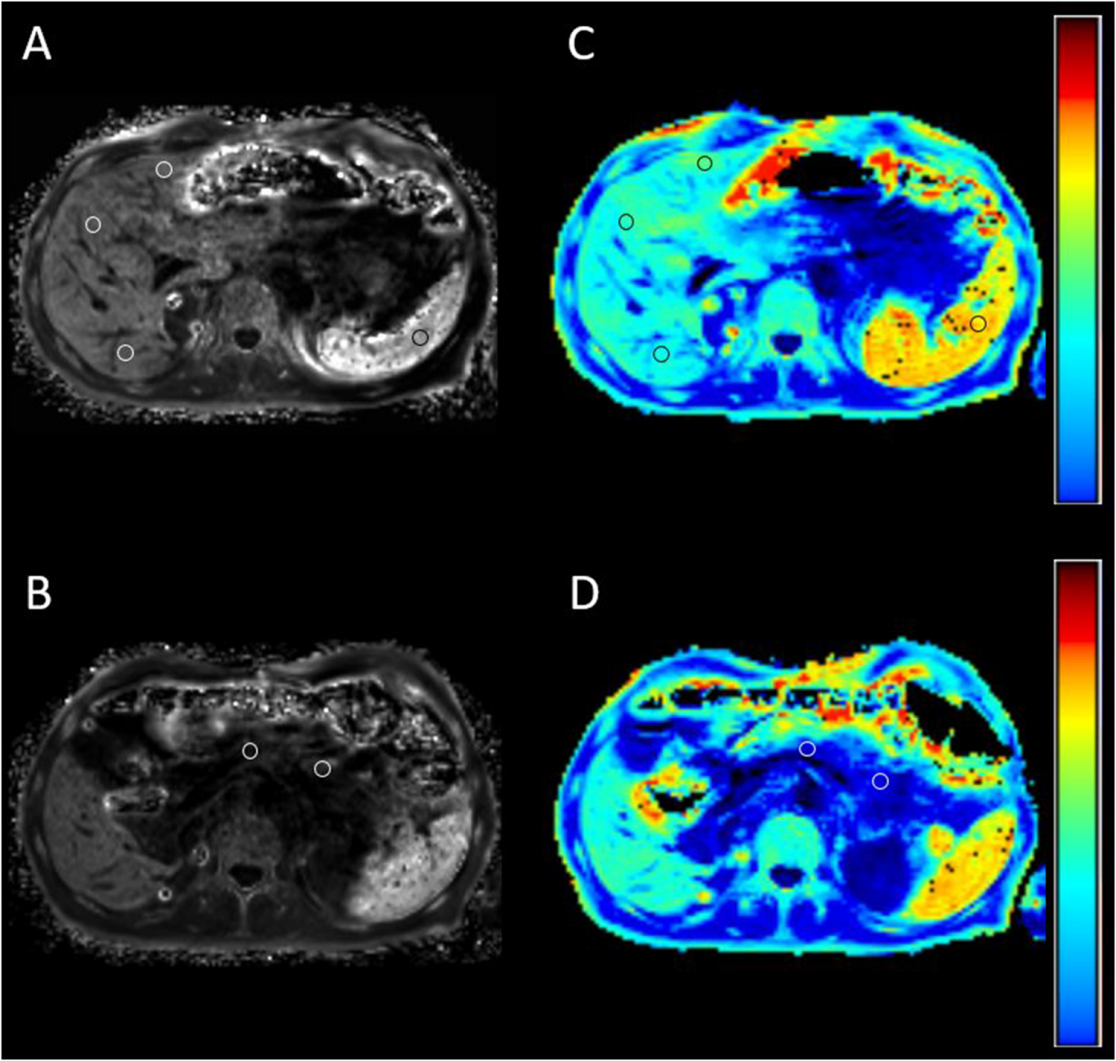
Splenic iron overload with central ROI placement on R2* maps of the 3D-qDixon sequence showing a mean R2* of 160 1/s (**A**) and a mean R2* of 186.6 1/s on a fat-saturated R2* relaxometry ME-GRE sequence (**C**). The images on the bottom reveal mean normal iron level in the pancreas using both methods. One ROI is positioned within the pancreatic corpus and one in the tail with a mean R2* of 37.05 1/s for the 3D-qDixon sequence (**B**) and 47.35 1/s for the ME-GRE sequence (**D**)

Figure 5 demonstrates a patient example with iron overload in the spleen and normal iron load in the pancreas using the two different methods.

## Discussion

In our study, we have shown that the commercial qDixon sequence, which is a 3D chemical shift imaging sequence, can apart from the liver also be reliably used to assess splenic and pancreatic iron overload.

The qDixon sequence and similar 3D chemical shift imaging sequences are only approved for imaging of the liver. They typically acquire a series of 6 gradient echoes and use a multi-fat peak model to obtain hepatic R2* and PDFF simultaneously from the obtained signals. This underlying multi-fat peak model, however, is usually derived from in vivo liver spectroscopy [12, 14] and thus, strictly speaking, only holds true for liver tissue. However, as the spleen and the pancreas are included in the acquired 3D volume of the upper abdomen, these two organs could be evaluated simultaneously in one examination. This would provide important additional information regarding differential diagnosis and the need for further imaging [3] whenever managing hyperferritinemia [7] and evaluating patients with suspected iron overload.

The algorithms used for R2* and PDFF parameter estimation from commercial 3D chemical shift imaging sequences use complex fitting of multi-spectral hepatic fat models and thus [13] necessitate prior knowledge of hepatic triglyceride spectra. Therefore, deviation of these spectra potentially induces quantification bias when performing measurements in organs other than the liver. Although Pezeshkian et al observed regional differences in fat composition between epicardial and subcutaneous adipose tissues [22], Reeder et al state that spectral positions of fat peaks are relatively constant across different types of fat and that differences between them probably have minimal influence on fat fraction measurements [23]. This is in agreement with Fukui et al [24] who found a significant correlation between histological pancreatic fat fraction and PDFF values obtained with a 3D chemical shift sequence which was also based on a hepatic multi-fat peak model. Hong et al [25] investigated the effects of varying six-peak triglyceride spectral models on PDFF assessment and demonstrated robustness of PDFF estimation across the biologically plausible range of triglyceride spectra over a wide range of different hepatic fat contents. Although confirming an increase of absolute estimation bias with higher PDFF, they underlined its small magnitude and therefore likely clinical insignificance. Moreover, they similarly found only minor bias in R2* estimation. This suggests reliable R2* quantification by 3D chemical shift imaging sequences also in organs other than the liver.

To date, using 3D chemical shift imaging sequences, only PDFF quantification in the spleen and the pancreas has been studied [25–29] mostly by comparing with MR spectroscopy (MRS) as a reference standard [30, 31]. 3D chemical shift imaging sequences have been used earlier to assess R2* in the spleen or the pancreas [26, 32], but to the best of our knowledge, so far, no study exists regarding the reliability of R2* values of these organs obtained with 3D multi-echo chemical shift imaging sequences. In our study, we compared R2* values obtained with the qDixon sequence for the pancreas and the spleen with values obtained with a 2D multi-gradient echo sequence (ME-GRE) which did not rely on prior knowledge of fat spectra but used simple magnitude fitting of a truncated exponential model. The used sequence applied fat suppression to improve the goodness-of-fit which might be reduced due to the confounding effect of fat. The used method closely corresponds to the biopsy-calibrated sequence used by Plaikner et al [33] who have shown very small differences between fat and non-fat suppression for R2* < 400 1/s which corresponds to the range of values observed in this study for the pancreas and the spleen.

Only modest agreement but strong correlation between R2* values of the compared methods was found for the pancreas (CCC = 0.714) while agreement was found to be excellent for the spleen (CCC = 0.934). In contrast, for the classification in iron overload or no iron overload based on R2* thresholds, strong agreement was found between both methods for the pancreas (overall agreement: 93.18%) as well as the spleen (overall agreement: 91.43%). The modest agreement for pancreatic R2* values might, among other reasons, be explained by the difference in pancreatic coverage of the used sequences. Whereas qDixon provides volume coverage with a slice thickness of 3.5 mm, pancreatic R2* maps for the used 2D ME-GRE sequence were acquired only with a single slice of 8 mm thickness. Poor slice positioning and partial volume effects thus most probably explain variability of individual R2* values. A similar effect was discussed by Coe et al [34] who obtained a modest level of agreement for PDFF values in the pancreas between a 3D chemical shift sequence and spectroscopy, stating that the “3D dimensionality” of the pancreas must be accounted for. In this context, for the pancreas potential measurement variability due to the heterogeneous shape of the organ, difficulty in delineating the contours, especially in the presence of atrophy or severe fat infiltration, and possible susceptibility artifacts due to adjacent intestinal gases [3] were already reported [35].

Unlike the pancreas, the spleen shows fatty infiltration only in very rare cases [26, 36] and usually has no histologically detectable fat. This would suggest that the particular multi-fat-peak model used for parameter estimation of 3D chemical shift imaging sequences only has limited effect, explaining the observed strong agreement for R2* values. For the spleen, Hong et al [26] found a slight overestimation (~ 2%) of PDFF values by 3D chemical shift -MRI compared to MRS which was explained as artifacts due to ghosting or aliasing or due to noise floor effects. It should be mentioned that they also investigated the correlation between splenic R2* and PDFF values but no comparison of R2* values between different methods was performed.

Finally, it must be noted that although for the liver a conversion of hepatic R2* values into hepatic iron concentration based on biopsy-based correlations is possible [37], such conversions do not currently exist for the pancreas and the spleen, because biopsy is either not feasible or not justifiable. In contrast to the liver, no splenic- and pancreatic-specific conversion factor based on biopsy is known until now. In some human studies, e.g., for the spleen, a calibration equal to the liver was assumed [38, 39], while other studies in mice showed significant differences between liver and spleen calibrations [8]. Therefore, future additional studies employing tissue samples are certainly necessary for calibration.

This retrospective study has some limitations: Supplemental clinical information of the included patients was not used for patient selection resulting in a heterogeneous group with only few patients with hepatic iron overload. Iron overload in the investigated organs was not confirmed by histology, because, as mentioned above, biopsy is not justified for the spleen or the pancreas. We did not correlate R2* values between the different organs, and we did not correlate with PDFF values, even though these data were provided by the qDixon sequence. We only focused on comparing the R2* values between our two MRI methods for the spleen and the pancreas, regardless of patient’s disease. For the liver, such comparisons have already been published before. Furthermore, also different iron distribution patterns between the organs were not evaluated and correlated with pathology. Iron distribution patterns play an important role in the differential diagnosis of iron overload disease [2, 40]; nevertheless, this was beyond the aim of our study.

In conclusion, our data show good agreement between R2* values obtained with a commercial qDixon sequence and a validated ME-GRE relaxometry method for spleen and pancreas. Therefore, the qDixon sequence, primarily intended for liver assessment, seems to be a reliable tool for the additional evaluation of these organs in the upper abdomen enabling an optimal diagnostic workflow for further differential diagnosis and patient management regarding iron status.

## Abbreviations

CCC: Concordance correlation coefficients
LIC: Liver iron concentration
LoA: Limits of Agreement
ME-GRE: 2D multi-gradient echo sequence
MR: Magnetic resonance
PDFF: Proton density fat fraction
qDixon: 3D chemical shift imaging sequence
ROI: Region of interest

## Declarations

### Guarantor

The scientific guarantor of this publication is Dr. Christian Kremser.

### Statistics and biometry

One of the authors has significant statistical expertise.

### Methodology

• retrospective

• observational

• performed at one institution

## References

[1] Aslan E, Luo JW, Lesage A (2021). MRI-based R2* mapping in patients with suspected or known iron overload. Abdom Radiol (NY).

[2] Weiss G (2010). Genetic mechanisms and modifying factors in hereditary hemochromatosis. Nat Rev Gastroenterol Hepatol.

[3] Noetzli LJ, Papudesi J, Coates TD, Wood JC (2009). Pancreatic iron loading predicts cardiac iron loading in thalassemia major. Blood.

[4] Henninger B, Alustiza J, Garbowski M, Gandon Y (2020). Practical guide to quantification of hepatic iron with MRI. Eur Radiol.

[5] Nairz M, Theurl I, Schroll A (2009). Absence of functional Hfe protects mice from invasive Salmonella enterica serovar Typhimurium infection via induction of lipocalin-2. Blood.

[6] Cairo G, Recalcati S, Montosi G, Castrusini E, Conte D, Pietrangelo A (1997). Inappropriately high iron regulatory protein activity in monocytes of patients with genetic hemochromatosis. Blood.

[7] Pietrangelo A, Corradini E, Ferrara F (2006). Magnetic resonance imaging to identify classic and nonclassic forms of ferroportin disease. Blood Cells Mol Dis.

[8] Hitti E, Eliat PA, Abgueguen E (2010). MRI quantification of splenic iron concentration in mouse. J Magn Reson Imaging.

[9] Franca M, Carvalho JG (2020). MR imaging assessment and quantification of liver iron. Abdom Radiol (NY).

[10] Meloni A, Positano V, Pistoia L, Cademartiri F (2022). Pancreatic iron quantification with MR imaging: a practical guide. Abdom Radiol (NY).

[11] Henninger B, Plaikner M, Zoller H (2021). Performance of different Dixon-based methods for MR liver iron assessment in comparison to a biopsy-validated R2* relaxometry method. Eur Radiol.

[12] Hamilton G, Yokoo T, Bydder M (2011). In vivo characterization of the liver fat (1)H MR spectrum. NMR Biomed.

[13] Hernando D, Kramer JH, Reeder SB (2013). Multipeak fat-corrected complex R2* relaxometry: theory, optimization, and clinical validation. Magn Reson Med.

[14] Kuhn JP, Hernando D, Munoz del Rio A (2012). Effect of multipeak spectral modeling of fat for liver iron and fat quantification: correlation of biopsy with MR imaging results. Radiology.

[15] Henninger B, Zoller H, Kannengiesser S, Zhong X, Jaschke W, Kremser C (2017). 3D multiecho Dixon for the evaluation of hepatic iron and fat in a clinical setting. J Magn Reson Imaging.

[16] Henninger B, Zoller H, Rauch S (2015). R2* relaxometry for the quantification of hepatic iron overload: biopsy-based calibration and comparison with the literature. Rofo.

[17] Breuer FA, Blaimer M, Heidemann RM, Mueller MF, Griswold MA, Jakob PM (2005). Controlled aliasing in parallel imaging results in higher acceleration (CAIPIRINHA) for multi-slice imaging. Magn Reson Med.

[18] R Development Core Team (2017) R: a language and environment for statistical computing. R Foundation for Statistical Computing, Vienna, Austria

[19] Martire R Reliability Coefficients. R package version 1.3.1. Available via https://CRAN.R-project.org/package=rel. Accessed March 12, 2019

[20] Zec S, Soriani N, Comoretto R, Baldi I (2017). High agreement and high prevalence: the paradox of Cohen’s kappa. Open Nurs J.

[21] Gwet KL (2008). Computing inter-rater reliability and its variance in the presence of high agreement. Br J Math Stat Psychol.

[22] Pezeshkian M, Noori M, Najjarpour-Jabbari H (2009). Fatty acid composition of epicardial and subcutaneous human adipose tissue. Metab Syndr Relat Disord.

[23] Reeder SB, Robson PM, Yu H (2009). Quantification of hepatic steatosis with MRI: the effects of accurate fat spectral modeling. J Magn Reson Imaging.

[24] Fukui H, Hori M, Fukuda Y (2019). Evaluation of fatty pancreas by proton density fat fraction using 3-T magnetic resonance imaging and its association with pancreatic cancer. Eur J Radiol.

[25] Hong CW, Mamidipalli A, Hooker JC (2018). MRI proton density fat fraction is robust across the biologically plausible range of triglyceride spectra in adults with nonalcoholic steatohepatitis. J Magn Reson Imaging.

[26] Hong CW, Hamilton G, Hooker C (2019). Measurement of spleen fat on MRI-proton density fat fraction arises from reconstruction of noise. Abdom Radiol (NY).

[27] Park CC, Hooker C, Hooker JC (2019). Assessment of a high-SNR chemical-shift-encoded MRI with complex reconstruction for proton density fat fraction (PDFF) estimation overall and in the low-fat range. J Magn Reson Imaging.

[28] Peterson P, Trinh L, Mansson S (2021). Quantitative (1) H MRI and MRS of fatty acid composition. Magn Reson Med.

[29] Weis J, Ahlstrom H, Korsgren O (2019). Proton MR spectroscopy of human pancreas allografts. MAGMA.

[30] Yokoo T, Shiehmorteza M, Hamilton G (2011). Estimation of hepatic proton-density fat fraction by using MR imaging at 3.0 T. Radiology.

[31] Hernando D, Sharma SD, Aliyari Ghasabeh M (2017). Multisite, multivendor validation of the accuracy and reproducibility of proton-density fat-fraction quantification at 1.5T and 3T using a fat-water phantom. Magn Reson Med.

[32] Idilman IS, Gumruk F, Haliloglu M, Karcaaltincaba M (2016). The feasibility of magnetic resonance imaging for quantification of liver, pancreas, spleen, vertebral bone marrow, and renal cortex R2* and proton density fat fraction in transfusion-related iron overload. Turk J Haematol.

[33] Plaikner M, Kremser C, Zoller H (2020). Evaluation of liver iron overload with R2* relaxometry with versus without fat suppression: both are clinically accurate but there are differences. Eur Radiol.

[34] Coe PO, Williams SR, Morris DM (2018). Development of MR quantified pancreatic fat deposition as a cancer risk biomarker. Pancreatology.

[35] Idilman IS, Yildiz AE, Karaosmanoglu AD, Ozmen MN, Akata D, Karcaaltincaba M (2022). Proton density fat fraction: magnetic resonance imaging applications beyond the liver. Diagn Interv Radiol.

[36] Forbes GB (1978). Splenic lipidosis after administration of intravenous fat emulsions. J Clin Pathol.

[37] Wood JC (2014). Use of magnetic resonance imaging to monitor iron overload. Hematol Oncol Clin North Am.

[38] Brewer CJ, Coates TD, Wood JC (2009). Spleen R2 and R2* in iron-overloaded patients with sickle cell disease and thalassemia major. J Magn Reson Imaging.

[39] Schwenzer NF, Machann J, Haap MM (2008). T2* relaxometry in liver, pancreas, and spleen in a healthy cohort of one hundred twenty-nine subjects-correlation with age, gender, and serum ferritin. Invest Radiol.

[40] Franca M, Marti-Bonmati L, Porto G (2018). Tissue iron quantification in chronic liver diseases using MRI shows a relationship between iron accumulation in liver, spleen, and bone marrow. Clin Radiol.

